# Hereditary Hemorrhagic Telangiectasia Associating Neuropsychiatric Manifestations with a Significant Impact on Disease Management—Case Report and Literature Review

**DOI:** 10.3390/life12071059

**Published:** 2022-07-15

**Authors:** Fabiola Sârbu, Violeta Diana Oprea, Alin Laurențiu Tatu, Eduard Polea Drima, Violeta Claudia Bojincă, Aurelia Romila

**Affiliations:** 1 Medical Department, Faculty of Medicine and Pharmacy, “Dunărea de Jos” University of Galati, 800216 Galati, Romania; sarbu_fabiola@yahoo.co.uk (F.S.); drima_edi1963@yahoo.com (E.P.D.); aurelia.romila@yahoo.com (A.R.); 2 “Elisabeta Doamna” Psychiatric Hospital, 800179 Galati, Romania; 3 “St. Apostle Andrei” Clinical Emergency County Hospital Galati, 800578 Galati, Romania; 4 Clinical, Medical Department, Dermatology, ReForm UDJ, Faculty of Medicine and Pharmacy, “Dunărea de Jos” University of Galati, 800216 Galati, Romania; 5 Dermatology Department, Clinical Hospital of Infectious Diseases Saint Parascheva, 800179 Galati, Romania; 6 Internal Medicine Department, Faculty of Medicine, “Carol Davila” University of Medicine and Pharmacy in Bucharest, 020021 Bucharest, Romania; vmbojinca@yahoo.com; 7 Department of Internal Medicine and Rheumatology, “Sf. Maria” Hospital, 011172 Bucharest, Romania

**Keywords:** hereditary hemorrhagic telangiectasia, Rendu–Osler–Weber syndrome, arteriovenous malformations, epistaxis, mental illness, neuropsychiatric, depression, anxiety

## Abstract

(1) Background: Genetic hereditary hemorrhagic telangiectasia (HHT) is clinically diagnosed. The clinical manifestations and lack of curative therapeutic interventions may lead to mental illnesses, mainly from the depression–anxiety spectrum. (2) Methods: We report the case of a 69-year-old patient diagnosed with HHT and associated psychiatric disorders; a comprehensive literature review was performed based on relevant keywords. (3) Results: Curaçao diagnostic criteria based the HHT diagnosis in our patient case at 63 years old around the surgical interventions for a basal cell carcinoma, after multiple episodes of epistaxis beginning in childhood, but with a long symptom-free period between 20 and 45 years of age. The anxiety–depressive disorder associated with nosocomephobia resulted in a delayed diagnosis and low adherence to medical monitoring. A comprehensive literature review revealed the scarcity of publications analyzing the impact of psychiatric disorders linked to this rare condition, frequently associating behavioral disengagement as a coping strategy, psychological distress, anxiety, depression, and hopelessness. (4) Conclusions: As patients with HHT face traumatic experiences from disease-related causes as well as recurring emergency hospital visits, active monitoring for mental illnesses and psychological support should be considered as part of the initial medical approach and throughout the continuum of care.

## 1. Introduction

Patients suffering from Rendu–Osler–Weber syndrome or hereditary hemorrhagic telangiectasia (HHT) are frequently diagnosed very late, as the manifestations require knowledge of this rare disease affecting 1 in 5000–8000 individuals globally [1,2]. HHT is an autosomal dominant genetic disorder, with ~90% of the patients associated with heterozygous mutations of ACVRL1 or ENG genes, while ~10% of cases also affect genes that encode components of the BMP9/ALK1 signaling pathway [2,3,4].

The mutations in HHT alter TGF-beta-mediated pathways in vascular endothelial cells, resulting in the manifestation of aberrant blood vessel development with extreme fragility and arteriovenous malformations (AVMs) [3,4,5]. The most prevalent clinical symptoms include epistaxis, which can be severe or even lethal in rare cases; telangiectasia (cutaneous blood vessels dilatations); and manifestations induced by AVMs or telangiectasia at the pulmonary, gastrointestinal hepatic, or cerebral level. These may lead to hemorrhages and anemia as well as other serious complications. Studies report that ~50% of HHT patients experience disabling or life-threatening complications, such as stroke or transient ischemic attacks, cerebral abscess, or heart failure [5,6].

A positive diagnosis of HHT is almost always clinical, based on the Curaçao diagnostic criteria established in 1999 [3,7,8], requiring at least three out of these four criteria to be met: spontaneous recurrent epistaxis, multiple telangiectasias, visceral involvement, and a relevant family history. If only two characteristics are present, the diagnosis is probable, and further tests or follow-up are necessary. The genetic tests are also available in some centers.

Concerning the alarming manifestations and scarce therapy options, and in relation to intrinsic pathophysiology, these patients may develop mental illnesses like anxiety, post-traumatic stress disorder, and depression [8,9]. Moreover, the presence of cerebral AVMs—although less frequent, identified in 10–20% of HHT cases [8,10,11]—could induce psychiatric manifestations depending on their localization, although these are very rarely mentioned in the available publications [6,7,11].

## 2. Materials and Methods

We report the case of a 69-year-old female patient suffering from HHT and associated severe depression, with episodes of anxiety significantly impacting her quality of life. A comprehensive literature review was performed based on relevant keywords, using PubMed and Google Scholar databases.

## 3. Results

The patient has been under clinical monitoring by an ad-hoc multidisciplinary medical team since February 2021. She recalls multiple episodes of epistaxis during childhood, spontaneously remitting at around 10 years old. She was symptom-free until 45 years of age, when she started to observe cutaneous telangiectasias and reported occasional nose bleeds, for which she rarely consulted her family physician. A severe epistaxis occurred in 2020, requiring emergency intervention; anemic syndrome was diagnosed and the medical team suspected HHT, recommending additional specialty examinations. She is highly anxious and refuses further investigations, and she presents herself to outpatient psychiatry complaining of unrest and depressive symptomatology, sleep disturbances, attention deficits, and emotional lability. She was diagnosed with mixed anxiety and depressive disorder (ICD-10 code F41.2 [12]), receiving a prescription for an antidepressant (sertraline 50 mg/day) and anxiolytic (alprazolam 0.25 mg/day), and it was also recommended that she undergo psychotherapy. The patient uses the medication intermittently upon symptom amelioration, and has been missing the advised periodic follow-ups.

At 63 years old, a nasal cutaneous ulcerative lesion brought the patient to a dermatologist, who identified a basocellular carcinoma, but also insisted on identifying diagnostic criteria for HHT, asking about hereditary background. In the presence of multiple telangiectasias, anamnesis of recurrent epistaxis, and a relevant family history (mother with similar cutaneous telangiectasias and retinal arteriovenous malformations, resulting in retinal scars with consequent decreased visual acuity; a sister with similar ocular manifestations; and father deceased at a young age after four successive strokes), the patient was diagnosed with HHT.

Mohs surgery was performed for the basocellular carcinoma, and further investigations related to HHT revealed a mild anemia (hemoglobin (Hb)—10.9 mg/dL, mean corpuscular volume (MCV)—76 fL with a normal interval of 80–96 fL, and iron deficit at 28 mg/dL). The results of an abdominal ultrasound were normal, except for a few renal cortical microcysts in her left kidney. Upon receiving detailed information on her newly diagnosed genetic disease, the anxiety symptoms reoccurred and the patient refused to undergo further imaging, fearing what it may discover.

Over the last 6 years, the patient presented several aggravated episodes of epistaxis (2–5 times/year) requiring medical assistance, which were treated for chemical/surgical cautery and/or hemostatic sponges; chronic anemia; hypertension (systolic blood pressure increased up to 180 mmHg); and other cardiac-related symptoms like palpitations and dyspnea, urinary symptoms due to a urethral polyp, three severe episodes of depression requiring hospitalization (10 to 21 days’ admittance), and recurrent headache associated with dizziness.

**Figure 1:**
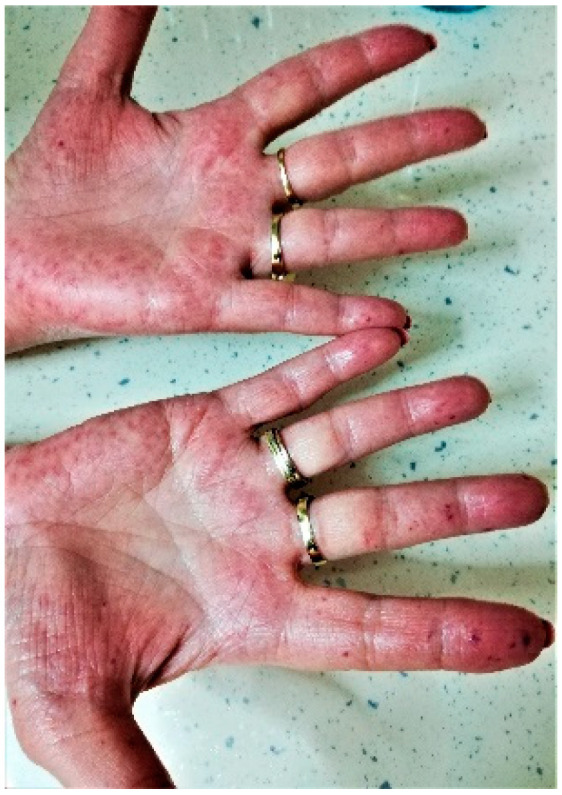
Multiple cutaneous telangiectasias on both hands—clinical presentation in February 2021.

**Figure 2:**
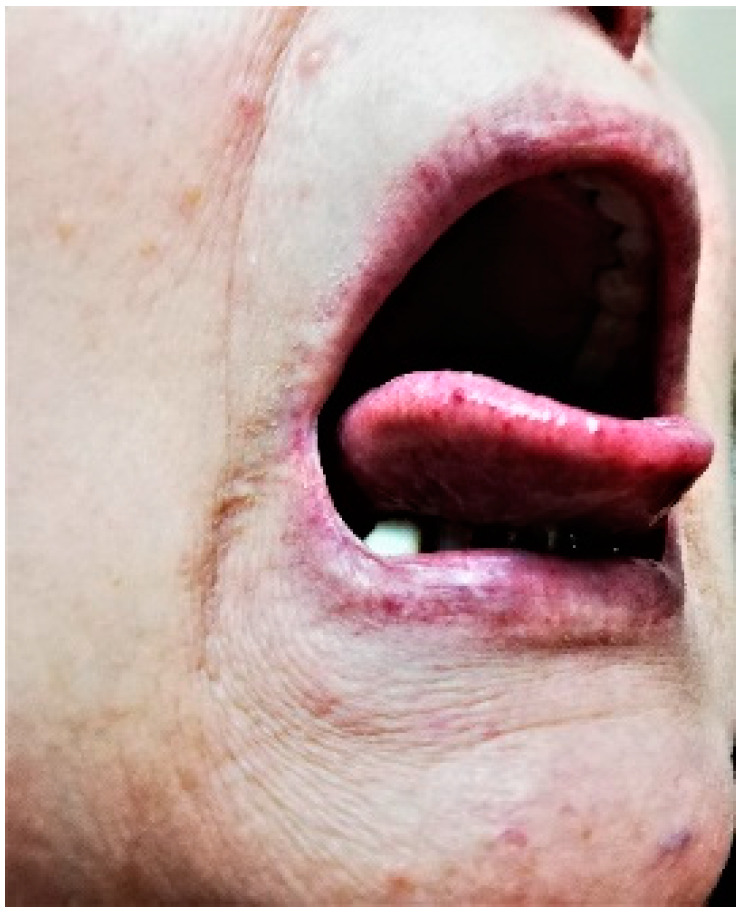
Mucosal telangiectasias at tongue and lips level—clinical presentation in February 2021.

**Figure 3:**
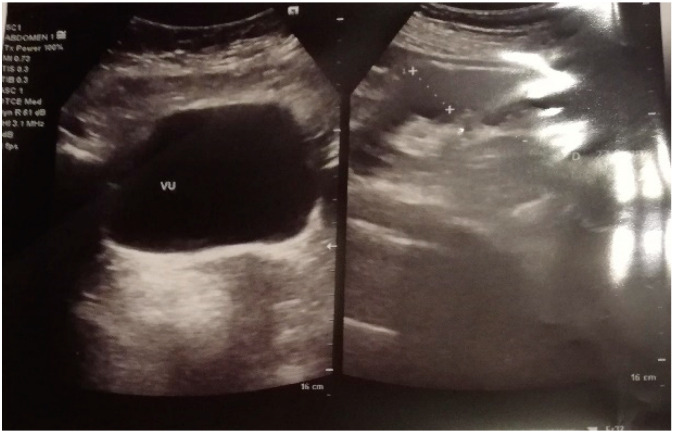
Ultrasound image of bladder, with minimal post-void residue—February 2021 (VU = urinary bladder).

**Figure 4:**
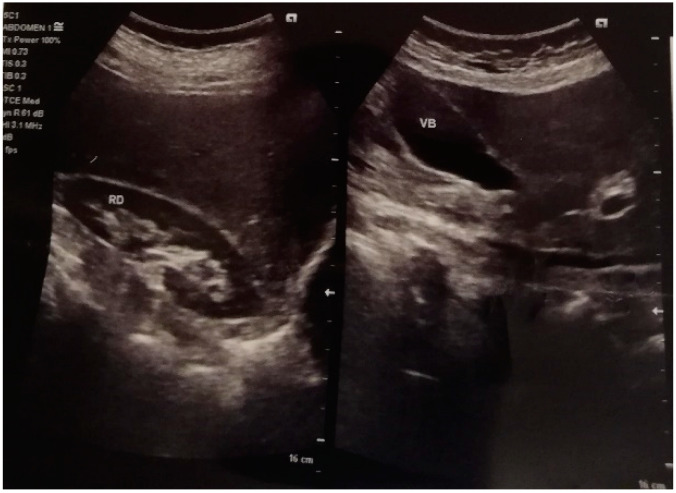
Ultrasound image of renal microcysts in the right kidney and normal image of biliary bladder—February 2021 (RD = right kidney; VB = gallbladder).

In February 2021, after another severe epistaxis that was treated at the emergency room by Merocel nasal packing, the patient was referred to our psychiatry clinic and agreed to a complex medical evaluation, which revealed the following:

1. Psychiatric evaluation:
  - Severe depression (scoring 22 when evaluated by Hamilton Depression Rating Scale (HAM-D) [13,14,15] and 23 by Geriatric Depression Scale (GDS) [16]);
  - Associated anxiety manifestations (evaluated by Hamilton Anxiety Rating Scale (HRSA)—score 27 [17,18];
  - Autolytic ideation, dysphoria, tendency to negative thinking with focus on self and the genetic disease, insomia, nosocomephobia, somatization;
  - Score of Global Assessment of Functioning Scale (GAFS) [19,20,21] of 40, corresponding to impairment in communication and reality testing, significant impact regarding family relations, thinking, and mood;
  - Amnestic Mild Cognitive Impairment (MCI) was determined using Mini Mental State Examination scale (MMSE [21,22,23,24,25,26])—score 23, Montreal Cognitive Assessment (MoCA) [21,24,27]—score 22.4, Clock Drawing Test [26,27], and Clinical Dementia Rating-Sum of Boxes (CDR-SOB) [22,23,27,28,29,30]—score 2.1.
2. Clinical check-up:
  - Moderate hypertension (154/85 mmHg) with a low patient adherence to recommended chronic therapy (candesartan 8 m twice daily, metoprolol 50 mg/day, and lacidipine 4 mg/day);
  - Blood tests: microcytic anemia (Hb 10.4 mg/dL, MCV 72 fL, iron deficit 29 µg/dL), hypercholesterolemia 338.9 mg/dL, with LDL cholesterol (low density lipoprotein) 263.77 mg/dL and HDL cholesterol (high density lipoprotein) 53.5 mg/dL, and normal hepatic and renal profile.
  - Epistaxis Severity Score (ESS) [31,32] of 4.13—moderate.
  - Severe generalized atherosclerosis, indicated by Doppler ultrasound of carotids, vertebral, and subclavian arteries showing 40–76% obstructions.
3. Dermatological exam: multiple mucocutaneous telangiectasias, especially on the hands, face, nostrils, and tongue (see Figure 1 and Figure 2), with seborrheic keratosis on her thorax, and a nasal postoperative scar without any regional sign of recurrence from the previous basocellular carcinoma.

4. Urologic evaluation: microscopic hematuria due to urethral mucosal ectropion, overactive bladder syndrome, ultrasound showing few bilateral renal cortical microcysts, and minimal bladder post-void residue (see Figure 3 and Figure 4).

5. Gastroenterology exam: normal function, lack of gastrointestinal bleeding, and no hepatic AVM identified.
6. Pneumology assessment: normal lung function and normal image in chest CT.

The patient became progressively anxious despite specific anxiolytic medication, refusing cerebral imaging and further assessment, so she was discharged after 6 days with a prescription and a recommended 6-month follow-up scheduled.

Because of COVID-19 pandemic restrictions and her own fear of hospital/medical care, the patient missed the routine check-ups, but more strictly followed the medical advice on therapy, especially as she observed an improvement in her mental state and sleep. Medication used for 13 months: tianeptine 12.5 mg three times daily, alprazolam 0.5 mg/day, zopiclone 7.5 mg/day, candesartan 8 m twice daily, bisoprolol 5 mg/day, lacidipine 4 mg/day, atorvastatin 20 mg/day, combination of rutoside 20 mg + ascorbic acid 50 mg three times/day plus supplementary vitamin C 1000 mg/day.

She presented in March 2022 with improved clinical and paraclinical parameters:

- Improved symptomatology and scoring for depression and anxiety (Hamilton Depression Rating Scale (HRSD) score 17, Geriatric Depression Scale (GDS) score 15, Hamilton Anxiety Rating Scale (HRSA) score 18, Global Assesment of Functioning Scale (GAFS) score 52);
- Lack of severe episodes of epistaxis (thus no emergency room visits);
- Epistaxis Severity Score (ESS) 2.14 (mild);
- no anemia and normal blood tests, except for persistent hypercholesterolemia [33] (total cholesterolemia 224 mg/dL, HDL cholesterol 164 mg/dL).

Again, the patient declined brain imaging, which is necessary in order to identify any possible cerebral AVMs, claiming that she would rather not know and fearing the certitude of a brain impairment. She admitted that the last 2 years during the COVID-19 pandemic added to her anxiety and distress of potential difficulties to receive emergency medical care if needed. As no AVMs were identified by the imaging, the patient could not be offered a therapy targeting the underlying pro-angiogenic pathophysiologic mechanism, as per current local protocols [34,35,36,37,38,39].

## 4. Discussion

Mental illness associated with hereditary hemorrhagic telangiectasia is not frequently reported in the literature, but patients often present with a significant impact on their health-related quality of life [1,6,7,40,41]. There are many possible reasons for a high prevalence of depression (88.7%), post-traumatic stress disorder (28.1%), and anxiety (43.9%) in individuals living with HHT [40,41,42].

One reason is related to the fear of death and frequent visits to the hospital emergency room, as well as the presence of recurrent bleeding. HHT is associated with a significant morbidity and mortality—some studies indicate a double versus normal mortality rate in patients aged <60 years [1,6,8,42,43]. There are reports of a bimodal distribution of mortality, age-related, with peaks at 50 years old and then from 60 to 80 years old. Generally, the mortality of HHT is generated by complications of AVMs, particularly in the brain, lungs, and gastrointestinal area. In our case, each bleeding episode and even the chronic anemia associated with recurrent hemorrhaging added to the anxiety and fear of hospital care [6,7,40,41,42].

Another problem with depressive symptomatology is the rarity of the disease and limited information available for patients, as well as the lack of curative or event-preventative therapies. Some HHT individuals have difficulties in social or working life, have troubles maintaining normal family relationships, and may become either obsessed with or otherwise reluctant to anything regarding their disease [6,40,43,44].

Patients with HHT may exhibit abnormal language development, learning difficulties, disinhibited behavior, or schizophrenia-like symptoms at younger ages [44,45], and later life reports include various psychiatric manifestations such as depressive or manic episodes, delusions, incoherence of speech, euphoric mood, inhibited behavior, poor social judgment, extreme anxiety, or impairment of different cognitive processes [45,46,47].

Neuropsychiatric symptomatology associated with HHT diagnosis could be a pathogenic feature resulting from the mechanism of the disease. Hypo-oxygenation of the neurons or microscopic cerebral embolism may occur from silent pulmonary arteriovenous malformations. The disturbed angiogenesis with the fragility of small vessels could generate brain dysfunction and different psychiatric manifestations depending on the location of these disturbances [3,5,9,45]. A recent report presents the positive outcomes of psychiatric pharmacotherapy combined with counseling and psychological interventions in a case of HHT with associated symptoms of depression and anxiety—the benefit of this patient-centered approach was evident [48].

For our patient, mental issues prevented a complete medical evaluation and caused a poor adherence to any recommended medical schedule. It is likely that some of the epistaxis episodes were related to poorly managed high blood pressure, in the context of her vessel frailty. A lack of evidence regarding brain AVMs is a problem in itself, excluding a possible surgical removal, not supporting anti-vascular endothelial growth factor (VEGF) therapy, and maintaining constant fear of a lethal hemorrhagic stroke [34,35,36,37,38,39].

Scientific evidence has connected chronic anemia to mental illnesses (such as depression, mood, or affectivity disorders), but also to a higher degree of impaired cognition as an age-related phenomenon [49,50,51,52,53] through various or concurrent mechanisms: cerebral hypoxia; iron deficiency with subsequent impaired myelin integrity; or deficient synthesis processes of some neurotransmitters—dopamine, serotonin, norepinephrine, or even genetic alterations found in hemoglobin deficiencies associated with certain mental disorders [51,52,53]. These correlations were not consistently reported by all prospective trials, but for HHT, such a connection between chronic anemia following frequent bleeding and neuropsychiatric manifestations cannot be ignored.

Some researchers indicated that the neurovascular manifestations show an age-related penetrance with an increased prevalence of cerebral manifestations over the lifespan [41,45]. A large number of patients suffering from HHT are able to identify one or more traumatic events related to their disease that caused psychological distress; therefore, the longer they live and the more bleeding episodes they experience, the more mentally affected they may be [47,54,55,56,57,58,59]. Our patient has a long history of hypertension and a poor therapy adherence; therefore, the causality connection between high blood pressure and anxiety, depression, or even an evolution towards dementia cannot be excluded [54,55].

Patients should receive updated information concerning their disease, and diagnostic as well as therapeutic options, so that they understand that access to a more efficient management of the disease would significantly improve their life expectancy and quality of life [60,61,62]. Patient support groups/patient associations are proven to be very effective in helping all rare disease patients, in general, to better cope with their illness; unfortunately, in our case, the patient did not agree to be referred to a rare disease organization and no specific HHT patient group was locally available. The current guidelines for the approach to HHT [10,63] and research evidence show a broadening spectrum of therapeutic solutions like well-known or repurposed drugs (including bevacizumab, propranolol, pazopanib, tacrolimus, and antithrombotic drugs) and new surgical techniques [64,65,66,67,68,69]. These would require more supporting evidence on efficacy and standardized algorithms for clinical use.

As for other chronic conditions, the COVID-19 pandemic has also impacted HHT patients and in particular the case presented here. Nevertheless, we noted the paucity of publications related to HHT care during the COVID-19 pandemic. A PubMed search using both “hereditary hemorrhagic telangiectasia/Rendu–Osler–Weber syndrome” and “COVID-19/coronavirus/SARS-CoV2” returned only 11 publications (search performed on 17 May 2022); a positive counterbalancing is constituted by the quality of these articles, including frameworks from The European Rare Disease Network for HHT, recommending approaches that augment the health and safety of HHT patients [63]. It was demonstrated that HHT individuals may develop paradoxical thromboembolic stroke from pulmonary AVMs, but also possess an inherent prothrombotic state because of disturbances in the regulation of coagulation at the endothelial surface [3,9]. They present higher levels of coagulation factor VIII (FVIII) than the general population, which correlates with thrombotic risk. Although subjects suffering from COVID-19 are at increased risk of thrombosis, and we have strong evidence that subjects with HHT often suffer from medical conditions that may negatively influence the clinical course of COVID-19 (like chronic anemia, heart failure, and pulmonary AVMs), recent data suggest that, actually, HHT patients may generally present milder forms of SARS-CoV2 infection [70,71,72,73,74,75,76]. Some preliminary data show a significant decrease in inflammatory cytokines detected in the HHT population—with differences lower than 50% for some cytokines like interleukines (ILs: IL-6, IL-1β, and IL12p40) and around 50% for Chemokine (C-C motif) ligand 20 (CCL20), Thrombospondin-1 (TSP-1), and Activin A [70,71,72,73,74,75,76]. If confirmed by larger studies, these HHT characteristics may explain some sort of protection against developing severe forms of COVID-19 [70,71,72,73]. Our patient does not recall presenting with specific COVID-19 symptoms, but she opted for SARS-CoV2 vaccination in complete schedule as soon as it became available (March 2021).

## 5. Conclusions

The presented case is highly relevant to illustrate the neuropsychiatric profile of Rendu–Osler–Weber syndrome and the significant impact that associated depression and anxiety may have on the management of the disease. These mental issues could prevent the patient from complete investigations and adherence to recommended therapeutic approaches—in our case, early signs of a ruptured cerebral AVM could possibly have been ignored, resulting in severe or lethal consequences.

Early identification and even active screening for neuropsychiatric manifestations in HHT could help patients better manage their symptoms and avoid developing PTSD after severe hemorrhagic episodes. Moreover, continuous education regarding their disease and recent advances in medicine could help them overcome depression and anxiety and allow them to adhere to medical recommendations and even therapy for associated comorbidities. An improved health-related quality of life would enable HHT patients to enjoy a fulfilled life and act more effectively when acute events occur.

## Data Availability

Data supporting the reported results can be obtained from the main authors upon reasonable request.
